# Changes in LH Pulsatility Profiles in Dairy Heifers During Exposure to Oestrous Urine and Vaginal Mucus

**DOI:** 10.1111/j.1439-0531.2012.01997.x

**Published:** 2012-12

**Authors:** K Nordéus, R Båge, H Gustafsson, L Söderquist

**Affiliations:** Department of Clinical Sciences, Swedish University of Agricultural SciencesUppsala, Sweden

## Abstract

Difficulty in observing oestrus is a problem for many dairy farmers performing AI. Finding ways to synchronize oestrous cycles or strengthen display of oestrus without hormonal treatments would be of great interest because many consumers object to the use of exogenous hormones on healthy animals. Modification of reproductive cycles through chemical communication has been reported in several species including cattle. LH is an important regulator of the follicular phase and could possibly be subject to pheromonal influence. This study focuses on the effect of volatile compounds from oestrous substances on LH pulsatility preceding the preovulatory LH surge in cattle. Four heifers of the Swedish Red breed were kept individually in isolation. Exposure to water during the control cycle (CC), and bovine oestrous urine and vaginal mucus during the treated cycle (TC), started simultaneously with induction of oestrus. Blood sampling at 15-min intervals started 37 h after administration of PGF_2α_ and continued for 8 h. Monitoring of reproductive hormones, visual oestrus detection and ultrasonographic examination of the ovaries continued until ovulation had occurred. The mean concentration of LH at pulse nadir was significantly higher during TC (2.04 ± 0.18 ng/ml) than during CC (1.79 ± 0.16 ng/ml), and peak amplitude was significantly higher during CC (Δ1.03 ± 0.09) than during TC (Δ0.87 ± 0.09). No other parameters differed significantly between the two cycles. We conclude that the difference in LH pulsatility pattern may be an effect of exposing heifers to oestrous vaginal mucus and/or urine and that the mechanism behind this needs further investigation.

## Introduction

Pheromones are chemical signals that mediate interactions between individuals of the same species. Releaser pheromones induce direct behavioural changes, while primer pheromones cause neuroendocrine changes (Karlson and Luscher 1959; Brennan and Keverne 2004). In some mammals, such as mice (Whitten 1956; Vanderlee and Boot 1955), rats (McClintock 1978), sheep (Radford and Watson 1957) and humans (McClintock 1971), exposure to conspecifics or their excreta has been shown to cause synchronization of reproductive cycles in females, an effect often attributed to pheromones. There is some evidence that the same might apply to heifers. Izard and Vandenbergh (1982) found that the degree of oestrous synchrony in a group of dairy heifers after PGF_2α_ injection was greater in animals exposed to cervical mucus from oestrous cows than in animals exposed to water.

A major problem for the dairy industry today is the declining fertility associated with increasing milk yields. Pregnancy rates in dairy cows has fallen annually by 1% and 0.45% in the United Kingdom and in the United States, respectively, during the last two decades of the 20th century (Royal et al. 2002), a trend that is also seen in other developed countries (Lucy 2001). Owing to the decline in fertility, there is a growing need for new reproductive management tools. In many countries, hormonal treatments are used. However, finding a way to synchronize oestrous cycles in a herd or strengthening the display of oestrus without using hormonal treatments would be advantageous because consumers of dairy products in many European countries object to the use of exogenous hormones on healthy animals. Hence, pheromonal manipulation of the oestrous cycle has the potential to be of great economic value.

Three possible mechanisms through which olfactory signals can affect the oestrous cycle were proposed by Aron in 1979: (i) by stimulating neuroendocrine structures controlling the corpus luteum, (ii) by changing the growth rate of the follicles and (iii) by inducing an ovulatory release of LH. Studies in women have shown that the LH pulse frequency (Shinohara et al. 2001), as well as the timing of the preovulatory LH surge (Stern and McClintock 1998), can be affected by exposure to axillary secretions from other women. When a ram is introduced to a group of ewes in anoestrus, pulsatile secretion of LH starts within minutes and there is a preovulatory surge of LH within 36 h (Signoret 1991). There is also evidence to suggest that a similar male effect exists in cattle (Rekwot et al. 2001; Tauck et al. 2010). Hence, the pulsatility pattern and the preovulatory surge of LH are of great interest when investigating the effects of putative pheromones.

In a recent study, we investigated whether exposure of heifers to certain substances previously shown to contain pheromones could affect their endocrinology, ovarian dynamics or behaviour during the oestrous cycle with focus on pro-oestrous and oestrous phases. The effect of the substances on the preovulatory LH surge was studied thoroughly (submitted for publication). The present study focuses on the effect of these substances on the pattern of the pulsatile secretion of LH preceding the preovulatory LH surge.

## Material and Methods

### Experimental animals

Four cyclic heifers of the Swedish Red breed were housed individually in separate, isolated rooms at the Swedish University of Agricultural Sciences (SLU), Uppsala, Sweden. The rooms were adjacent and located pair-wise, and each room had a separate entrance. The heifers could not see each other, but they shared the same ventilation system and could, to some extent, hear each other. Changing of clothes (coats, boots and head wear) and washing of hands were mandatory before entering each room. The heifers were kept in tie stalls, where they were fed hay *ad libitum* and limited amounts of concentrate. The animals had been part of an antecedent study. Housing and personnel for the duration of this study were kept unchanged. The average age of the animals was 18.4 ± 0.8 months (mean ± SD, range 17.8–19.6 months) at the start of the experiment. The average body weight at the end of the experiment was 411.5 ± 33.7 kg (mean ± SD, range 368–448 kg), which is considered normal for heifers of this breed and age. The protocol for oestrus synchronization in the experimental heifers, previously used to assess LH pulse parameters (Morris et al. 2011), included an intramuscular injection of 5 ml (21 μg) of GnRH (buserelin acetate, Intervet, Boxmeer, the Netherlands) followed by an intramuscular injection of 2 ml (0.5 mg) of PGF_2α_ (cloprostenol sodium, Intervet) 7 days later during early midluteal phase.

### Donor animals

Vaginal mucus and urine were collected from cyclic heifers and cyclic, non-lactating cows of the Swedish Red and the Swedish Holstein breeds housed at SLU. Different individuals to the recipients were used as donors. The animals were housed in conventional tie stalls and fed hay *ad libitum*. In the donor animals, oestrus was induced by two intramuscular injections of 2 ml (0.5 mg) of PGF_2α_, administered 11 days apart.

### Vaginal mucus and urine

Collection of urine and vaginal mucus from the donor animals started approximately 48 h after the last injection of PGF_2α_, and only if there was at least one follicle larger than 12 mm present in the ovaries. The collection continued until ovulation. During this period, the animals’ ovaries were monitored with transrectal ultrasound, using portable ultrasound (Agroscan ALR 757; ECM, Angoulème, France) with a 7.5 MHz linear rectal probe, and visual oestrus detection was performed at least twice daily. Time of ovulation was determined as the midpoint between the last observation of the ovulatory follicle and the first observation of the ovulated ovary. Urination was induced, once during the 48 h, by manual stimulation of the perineum, and the urine was collected in a glass vial. It was then poured into polypropylene vials and stored at −80°C. The vaginal mucus was collected using tampons made of gauze and cotton string that were handmade for the purpose. The tampons were placed in the cranial vagina through a speculum and left there for 1–2 h before they were removed and stored in plastic vials at −80°C. In total, vaginal secretion was collected from six animals and urine from four animals. In relation to the ovulation, the substances collected were classified as pro-oestrus, oestrus or post-oestrus. The classification was made based on how long before ovulation the samples were collected and the signs of oestrus (lordosis, restlessness, oestrous vaginal discharge, and swelling and hyperaemia of the external genitalia) displayed by the donor animal at that time. The substances collected earlier than 48 h before ovulation or after ovulation were discarded. Of the substances used for exposure in the experiment, 10 samples were considered to be pro-oestrus, 15 oestrus and 7 post-oestrus.

### Experimental design

**Fig. 1:**
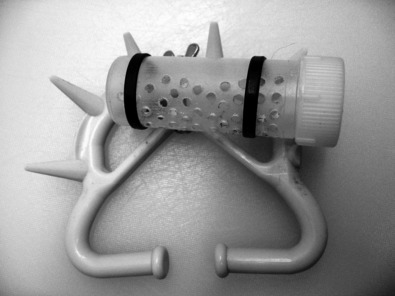
Nose ring and tampon

The exposure of the experimental heifers to the substances started simultaneously with the administration of PGF_2α_. The animals were fitted with a non-invasive nose ring intended to prevent unwanted suckling in calves (Cattle Weaner Müller, Albert Kerbl GmbH, Buchbach, Germany). During the control cycles, water was applied to clean tampons. During the treatment cycles, the vaginal mucus was presented to the animals on the cotton tampons used for collection, while the urine was applied to clean tampons. The tampons were placed in plastic cassettes attached to the nose ring (Fig. 1).

**Fig. 2:**
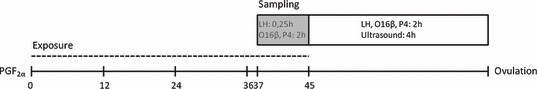
Experimental design. Exposure started simultaneously with the administration of PGF_2α_, and the substances were replaced every 12 h. Continuous blood sampling started 37 h after PGF_2α_ and continued until ovulation. During the first 8 h, samples were taken every 15 min for LH and every second hour for progesterone and oestradiol. Thereafter, samples were taken every second hour for all three hormones, and the ovaries were examined with ultrasonography every 4 h, simultaneously with visual oestrus detection

The tampons were replaced with fresh ones at 12-h intervals. The animals were also fitted with an indwelling silicone tube (SEDAT, Irigny, France) in the jugular vein the day before the intensive blood sampling, which started 37 h after the administration of PGF_2α_. The animals were bled every 15 min for 8 h after which the nose rings were removed. The blood sampling continued every second hour until ovulation had occurred. Ultrasonographic examination of the ovaries and visual oestrus detection was performed once daily after administration of PGF_2α_ and every 4 h after onset of pro-oestrus until ovulation. The onset of oestrus was defined as the midpoint between the last observation when no lordosis was recorded and the first observation when the animal displayed lordosis. Conversely, the end of oestrus was the midpoint between the last observation of lordosis and the following oestrus detection. This definition of oestrus in tie stalls is consistent with standing to be mounted by a bull (Gustafsson et al. 1986). The same procedure was carried out on each individual for both control substance (water) and test substances (both urine and vaginal mucus). Hence, each animal served as its own control (Fig. 2). At the end of the experiment, all animals were inseminated and sent to a research farm with commercial dairy production. Fertility data for the animals were later collected from the farm records.

All animal experiments described in this article were carried out in accordance with EC Directive 86/609/EEC and have been approved by the Uppsala regional ethical committee (approval no. C160/6).

### Follicular dynamics and hormonal profiles in the experimental animals

Ultrasonographic examinations of the ovaries were made using the same portable ultrasound as aforementioned, now fitted with a digital recording device (PMP-100, Sigmatek, France). Each examination was recorded as a digital file for retrospective analysis of the follicular dynamics. Starting the day before the intensive blood sampling, one ultrasound examination per animal each day was selected for the retrospective analysis. The follicles in both ovaries were divided into three categories (small <7 mm, medium 7–10 mm and large >10 mm), and the total number of follicles in each group was recorded. The ovulatory follicle was traced back to the first ultrasound or, if it developed later, to when it attained medium size. The mean growth rate per day from either the first ultrasound examination or entry into the medium class to ovulation was then calculated.

To obtain the pattern of the LH pulsatility preceding the preovulatory LH surge, blood samples taken every 15 min were assayed for LH. Blood samples taken at 2-h intervals between 34 and 14 h before ovulation were also analysed for LH, to identify the preovulatory LH surge. From the beginning of the intensive sampling for LH until 14 h before ovulation, blood samples were taken and assayed for progesterone and oestradiol-17β at 2-h intervals. The blood samples were collected into heparinized glass tubes (Venoject; Terumo Europe N. V., Leuven, Belgium) and centrifuged at 3000 × ***g*** for 10 min. The plasma was then removed and stored in plastic vials at −20°C until analysis.

Plasma concentrations of LH were determined using a double antibody radioimmunoassay (RIA) procedure, as described previously (Ayad et al. 2007). The minimum detection limit of the LH-RIA technique was 0.45 ng/ml. The intra- and interassay coefficients of variation (CVs) were 5.1% (6.9 ± 0.4 ng/ml) and 11.8% (6.6 ± 0.8 ng/ml), respectively. Progesterone concentrations were determined by a direct RIA method without extraction, as previously described in detail (Lopez-Gatius et al. 2007). The minimum detection limit of the P4-RIA technique used was 0.15 ng/ml and intra- and interassay CVs of 13% (2.6 ± 0.4 ng/ml) and 19% (2.7 ± 0.5 ng/ml), respectively. Plasma concentrations of oestradiol-17β were determined using a ^125^I RIA previously validated for bovine plasma (Sirois and Fortune 1990). The minimum detection limit was 4.1 pm, and the intra- and interassay CVs were 22.9 and 13.4% (5.5 pm), 4.5 and 16.6% (49.4 pm) and 7.8 and 14.5% (148.5 pm), respectively.

### Pulse analysis

Characteristics of the LH pulsatility were determined using a modified version of the ‘Pulsar’ algorithm developed by Merriam and Wachter (1982), which has been modified and adapted for the Apple Macintosh computer (‘Munro’, Zaristow Software, Haddington, UK). The G parameters were set at 3.799 (G1), 2.597 (G2), 1.900 (G3), 1.500 (G4) and 1.200 (G5). The Baxter parameters were 0.06600 (B1), 0.02500 (B2) and 0.00039 (B3). The analysis rendered number, amplitude, nadir concentration and area of the peaks.

### Statistical analysis

A marginal generalized estimating equations (GEE) linear regression model (Liang and Zeger 1986) with treatment as factor was estimated for each LH endpoint. An exchangeable correlation structure was used to take into account the dependencies between repeated peaks/measurements within heifers. Total production of each reproductive hormone was calculated as area under the curve (AUC), using the trapezoidal rule. The difference between control and treatment with regard to several characteristics of the oestrous cycle was tested using a paired *t*-test.

## Results

**Fig. 3:**
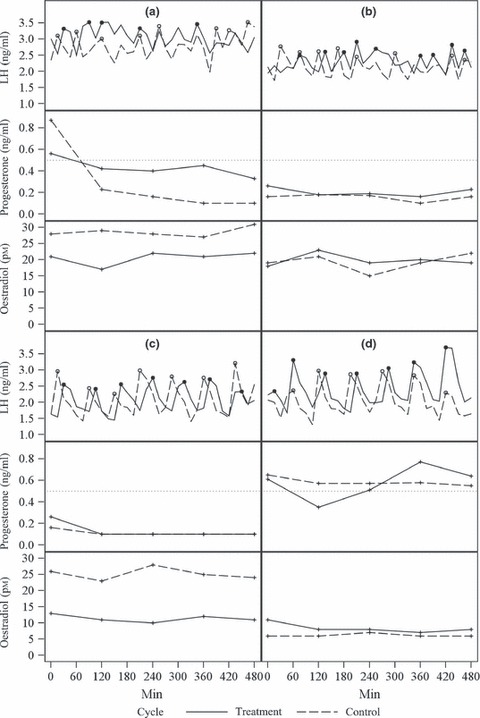
Hormonal profiles for animals (a–d), starting 37 h after administration of PGF_2α_. Circles mark peak values. The dotted lines show the baseline levels of progesterone (≤0.5 ng/ml)

The administration of PGF_2α_ occurred between days 5 and 14 of the oestrous cycle (day 1 = ovulation) before the control cycle and between days 9 and 11 before the treatment cycle. The mean concentration of LH at nadir was significantly higher (p < 0.001) during the treatment cycle (2.04 ± 0.18 ng/ml) than during the control cycle (1.79 ± 0.16 ng/ml). However, the mean peak concentrations of LH did not differ significantly between the control and treatment cycles (2.83 ± 0.13 and 2.92 ± 0.17 ng/ml, respectively). The peak amplitude was significantly higher (p = 0.001) during the control cycle (Δ1.03 ± 0.09) than during the treatment cycle (Δ0.87 ± 0.09). Neither the mean area of the peaks (27.60 ± 4.42 and 25.59 ± 3.02 ng/ml for control and treatment cycle, respectively) nor the mean interval between peaks (60.86 ± 2.02 and 58.61 ± 3.68 min for control and treatment cycle, respectively) differed significantly between the two cycles, indicating that there was no difference in total release of LH between treatment and control. The mean numbers of peaks during the 8-h sampling period were 7.3 and 7.0 for control and treatment, respectively, and this difference between treatments was not significant. Least squares means (LS-means) and standard error of the mean (SEM), from the marginal model, are presented for each treatment in Table 1, and the LH pulsatility profiles are illustrated in Fig. 3.

**Table 1:** LS-means (±SEM) for LH pulse characteristics from blood sampling intervals of 15 min for 8 h in four heifers during control and treatment oestrous cycles

Endpoint	Control	Treatment	p-value
Mean no. pulses: (min to max)	7.3 (6–8)	7.0 (5–9)	N.S.
Mean concentrations: (ng/ml)			
Amplitude	1.03 (±0.09)	0.87 (±0.09)	0.001
At nadir	1.79 (±0.16)	2.04 (±0.18)	<0.001
At peak	2.83 (±0.13)	2.92 (±0.17)	0.375
Area	27.60 (±4.42)	25.59 (±3.02)	0.383
Peak interval (min):	60.86 (±2.02)	58.61 (±3.68)	0.508

**Fig. 4:**
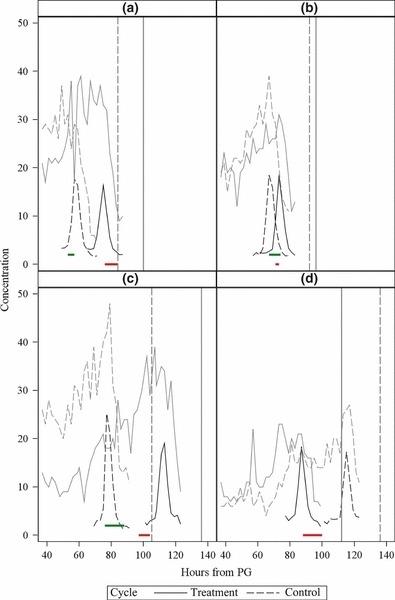
Oestradiol (grey) and LH surge (black) concentrations, duration of oestrus (horizontal lines, treatment = red, control = green) and ovulation (vertical lines) in animals (a–d) following administration of PGF_2α_

Oestradiol and LH concentrations, oestrus duration and ovulation for all animals following the administration of PGF_2α_ are presented in Fig. 4. The difference between control and treatment with regard to several characteristics of the oestrous cycle was tested using a paired t-test, and the results are presented in Table 2. None of these differences were significant. The mean total production of progesterone during the intensive blood sampling did not differ between the two cycles (2.2 and 2.6 ng/ml for control and treatment cycle, respectively). In all animals, except during the control cycle in animal D, basal levels of progesterone [<0.5 mg/ml (Bosu et al. 1981)] were reached before or during the first 2 h of the intensive blood sampling. In animal D, basal levels of progesterone were not reached until 24 h later during the control cycle.

**Table 2:** Influence of treatments on selected characteristics of the oestrous cycle in animals A–D

	A CTRL	A TRT	B CTRL	B TRT	C CTRL	C TRT	D CTRL	D TRT	CTRL Mean	TRT Mean	
Progesterone											
Concentration at PGF_2α_ adm. (ng/ml)	3.8	6.1	4.6	9.9	8.7	6.1	5.0	9.3	5.5	7.8	N.S.
Total production ‘Window’^1^ (ng/ml)	2.0	3.4	1.2	1.6	0.9	1.0	4.6	4.6	2.2	2.6	N.S.
Time ‘Window’^1^ to basal level (min)	120	120	0	0	0	0	1445	125	391.3	61.3	N.S.
Total production (ng/ml)	5.4	14.7	7.2	7.2	6.6	10.1	61.7	36.5	20.2	17.1	N.S.
Follicular dynamics Mean no of follicles/day											
<7 mm	9.3	20.5	5.8	28.0	22.8	21.8	28.8	21.0	21.7	22.8	N.S.
7–10 mm	0.0	0.5	0.0	0.0	0.6	0.7	1.0	2.2	0.4	0.8	N.S.
>10 mm	1.0	0.5	1.0	1.0	1.4	1.7	0.7	0.2	1.0	0.8	N.S.
Growth rate from >7 mm to ov. (mm/day)	1.0	1.0	1.2	1.5	0.6	0.5	1.5	2.5	1.1	1.4	N.S.
Time PGF_2α_ adm. to ovulation (h)	84	100	92	96	105	136	136	112	104.3	111.0	N.S.
Oestradiol											
Total production ‘Window’^1^ (pm)	227	163	151	161	203	90	50	66	157.8	120.0	N.S.
Max. concentration (pm)	37	39	39	31	48	39	27	23	37.8	33.0	N.S.
Time ‘Window’^1^ to max. concentration (h)	12	24	30	36	42	70	80	36	41.0	41.5	N.S.
Time from max. conc. to ovulation (h)	35	38	25	24	26	30	19	39	26.3	32.8	N.S.
Total production (pm)	804	1302	1017	1017	1500	1705	1099	834	1105.0	1214.4	N.S.
Preovulatory LH peak											
Peak concentration (ng/ml)	17.7	16.4	18.5	18.3	25.2	19.0	17.3	18.3	19.7	18.0	N.S.
Peak to onset of oestrus (h)	−4	1	0	−1	0	−15	–	1	−1.3	−3.5	N.S.
Peak to ovulation (h)	27	24	25	24	28	24	21	25	25.3	24.3	N.S.
Total production (ng/ml)	142.5	131.6	142.6	133.5	159.8	150.3	137.5	141.5	145.6	139.2	N.S.

1 ‘Window’ = intensive sampling period, 37–45 h after PGF_2α_

The pregnancy rate after the first insemination was 100%, and three of the animals gave birth to live calves.

## Discussion

The aim of this study was to investigate whether exposure to bovine oestrous vaginal mucus and urine previously shown to contain bioactive volatile compounds could alter the LH pulsatility pattern preceding the preovulatory LH surge in heifers. The exposure to control and test substances started simultaneously with the administration of PGF_2α_, and continuous blood sampling started 37 h later. The reason that PGF_2α_ was administered during a greater time span before the control cycle (days 5–14) than before the treatment cycle (days 9–11) was that the degree of synchrony among the animals before initiation of the synchronization protocol differed slightly. However, for both control and treatment cycles, the midpoint of the time span was similar, but the interval was greater for the control cycle. The fact that the dispersion of the LH data is similar for both control and treatment cycles indicates that the differences seen in LH pulsatility pattern were not caused by this difference in time span. The mean LH pulse frequency of 0.91 and 0.88 pulses/h for control and treatment, respectively, is similar to the frequency of 0.76 pulses/h reported by Morris et al. (2008), using the same protocol with GnRH and PGF_2α_. All animals became pregnant after the first insemination, and three of four animals gave birth to live calves, indicating that the animals were in good reproductive health throughout the study.

In the present study, we found that nadir concentrations of LH were significantly higher and peak amplitudes significantly lower during the treatment cycle than during the control cycle, but that peak concentrations were the same. Ginther et al. (2011) showed that LH concentrations at pulse nadirs were greater and pulse amplitude lower during the luteolytic than during the pre-luteolytic period, whereas peak concentrations did not differ between periods. However, the end of the luteolytic period was defined as the time when the progesterone concentration decreased to 1 ng/ml (Ginther et al. 2010). In the present study, the progesterone concentrations in all animals were ≤0.9 ng/ml at the onset of the intensive sampling period and should consequently be considered as post-luteolytic. Hence, the difference in nadir concentrations and amplitude between control and treatment cycles in this study cannot be explained by a faster onset of luteolysis during treatment cycles than during control cycles, even though the pattern of the LH pulsatility is consistent with such an interpretation.

Rahe et al. (1980) examined the LH pulsatility pattern during three different stages of the bovine oestrous cycle. During the preovulatory surge, LH pulsatility resembled that of the early luteal phase, which was characterized as high frequency (20–30 pulses/24 h) and low amplitude (Δ0.3–1.8 ng), while midluteal phase was classified as high amplitude and low frequency. Changes in concentrations of steroidal hormones were suggested as the mechanism behind this. LH release is suppressed by administration of exogenous progesterone, whereas continuous administration of oestradiol at concentrations similar to that of the follicular phase through implants enhances mean concentration of LH as a consequence of higher pulse amplitude (Rathbone et al. 2001). Contrary to expectations given the difference in LH pulsatility pattern between the two treatments in this study, the mean total production of progesterone and oestradiol did not differ between the two cycles during the intensive sampling period. One reason for this could be that the limited number of animals prevents the detection of such a difference. In one of the animals, basal levels of progesterone during the control cycle were reached approximately 24 h later than during all other cycles in all animals. The possibility that this animal may have been in an earlier stage than the other animals during the intensive blood sampling could have influenced the data. However, the same pattern with high nadirs and low amplitudes was seen in all animals during the treatment cycle. Also, during the treatment cycle, this animal did reach basal levels of progesterone at the normal time, but the levels fluctuated and several samples thereafter reached suprabasal levels (i.e. >0.5 ng/ml). Hence, it is not likely that this deviant animal influenced the results.

Exposing ewes to acute psychosocial stress (Dobson et al. 1999; Breen et al. 2007) or cattle to negative energy balance (Butler and Smith 1989) has a negative effect on the pulsatile secretion of LH. However, this is not a likely explanation for the difference in LH pulsatility pattern between the two treatments in this study, because housing conditions and handling of animals were identical during both cycles.

The experimental set-up of the current study was unique in that the heifers were managed in conventional barn environment although housed in isolation. Given the laborious and time-consuming experimental design, the use of a large number of animals was not possible. Unfortunately, the small number of animals may have prevented detection of underlying mechanisms that could explain the difference between the two treatments. Also, even though the pulsatile secretion of LH during the bovine oestrous cycle has been thoroughly investigated in the past, the exact details of the post-luteolytic pulsatility pattern remain unknown. In the light of the limited evidence for bovine olfactory communication between females previously presented, the results of the present pilot study are very interesting. However, it needs to be repeated on a larger number of animals, and the post-luteolytic LH pulsatility pattern needs to be investigated in more detail.

We conclude that the difference in LH pulsatility pattern seen in the present study, with greater nadir concentrations and lower amplitudes during the treatment cycle, is possibly an effect of exposure of heifers to vaginal mucus and/or urine from other oestrous females and that the mechanism behind this needs further investigation.
